# Silencing of the long non-coding RNA *LINC00265* triggers autophagy and apoptosis in lung cancer by reducing protein stability of SIN3A oncogene

**DOI:** 10.32604/or.2023.030771

**Published:** 2024-06-20

**Authors:** XIAOBI HUANG, CHUNYUAN CHEN, YONGYANG CHEN, HONGLIAN ZHOU, YONGHUA CHEN, ZHONG HUANG, YULIU XIE, BAIYANG LIU, YUDONG GUO, ZHIXIONG YANG, GUANGHUA CHEN, WENMEI SU

**Affiliations:** 1 Department of Pulmonary Oncology, Affiliated Hospital of Guangdong Medical University, Zhanjiang, 524001, China; 2 Department of Thoracic Surgery, Affiliated Hospital of Guangdong Medical University, Zhanjiang, 524001, China; 3 Department of Orthopedics, Affiliated Hospital of Guangdong Medical University, Zhanjiang, 524001, China; 4 Guangdong Provincial Key Laboratory of Autophagy and Major Chronic Non-Communicable Diseases, Affiliated Hospital of Guangdong Medical University, Zhanjiang, 524001, China

**Keywords:** *LINC00265*, Autophagy, Lung adenocarcinoma (LUAD), Cancer progression, Switch-independent 3a (SIN3A)

## Abstract

**Background:**

Long non-coding RNAs are important regulators in cancer biology and function either as tumor suppressors or as oncogenes. Their dysregulation has been closely associated with tumorigenesis. *LINC00265* is upregulated in lung adenocarcinoma and is a prognostic biomarker of this cancer. However, the mechanism underlying its function in cancer progression remains poorly understood.

**Methods:**

Here, the regulatory role of *LINC00265* in lung adenocarcinoma was examined using lung cancer cell lines, clinical samples, and xenografts.

**Results:**

We found that high levels of *LINC00265* expression were associated with shorter overall survival rate of patients, whereas knockdown of *LINC00265* inhibited proliferation of cancer cell lines and tumor growth in xenografts. Western blot and flow cytometry analyses indicated that silencing of *LINC00265* induced autophagy and apoptosis. Moreover, we showed that *LINC00265* interacted with and stabilized the transcriptional co-repressor Switch-independent 3a (SIN3A), which is a scaffold protein functioning either as a tumor repressor or as an oncogene in a context-dependent manner. Silencing of SIN3A also reduced proliferation of lung cancer cells, which was correlated with the induction of autophagy. These observations raise the possibility that *LINC00265* functions to promote the oncogenic activity of SIN3A in lung adenocarcinoma.

**Conclusions:**

Our findings thus identify SIN3A as a *LINC00265*-associated protein and should help to understand the mechanism underlying *LINC00265*-mediated oncogenesis.

## Introduction

Lung cancer is one of the most common malignant tumors and represents the leading cause of cancer-related deaths worldwide, with non-small cell lung cancer (NSCLC) accounting for 85% of lung cancer cases [1,2]. Despite advances in diagnosis and combined treatments, the prognosis of lung cancer patients remains unsatisfied. The 5-year survival of lung cancer is about 19% [3]. Therefore, there is an urgent need to improve the understanding of this cancer and to identify early diagnostic biomarkers and new therapeutic targets.

There is increasing evidence suggesting that long non-coding RNAs (lncRNAs) play important roles in lung cancer development by acting as both tumor suppressors and oncogenes [4]. Many lncRNAs are frequently dysregulated in cancers and associated with cancer progression [5,6]. Thus, they are promising biomarkers and therapeutic targets for lung diseases [7]. *LINC00265* was first identified in lung adenocarcinoma (LUAD) as an upregulated lncRNA and a prognostic biomarker of this cancer [8], suggesting a potentially important role of this lncRNA in cancer development. Indeed, *LINC00265* has been shown to promote cell proliferation and viability in a number of cancers by interacting with other non-coding RNAs and key signaling pathways such as Epidermal Growth Factor (EGF) and Wnt/β-catenin. It is upregulated in colorectal cancer (CRC) and associated with poor prognosis [9–11]. Higher levels of *LINC00265* were also found in the serum of patients with acute myeloid leukemia (AML), which is correlated with lower overall survival rate because *LINC00265* contributes to migration and invasion of tumor cells by regulating PI3K/AKT and signal transducer and activator of transcription 3 (STAT3) signaling [12–14]. Therefore, circulating *LINC00265* can be used as a biomarker for diagnosis and therapeutic monitoring of AML [15]. However, the mechanisms underlying *LINC00265* function in LUAD progression remain elusive.

The transcriptional corepressor SIN3A is a member of the SIN3 family and acts as a scaffold protein in the histone deacetylase (HDAC) complex. It can function either as a tumor suppressor or as an oncogene, depending on its biochemical interaction with specific protein partners, genetic interaction with other genes or regulation of target genes [16–18]. It has been shown that SIN3A inhibits cell invasion and is downregulated in cancers, thus functioning as a tumor suppressor [19,20]. Recently, there is also evidence indicating that SIN3A promotes the oncogenic potential of STAT3 and represses the transcription of tumor suppressor genes; interfering with its expression leads to cell death of anaplastic large-cell lymphoma through transcriptional activation of tumor suppressor genes [21].

In this work, we investigated the functional role of *LINC00265* in regulating LUAD progression. Using clinical samples and NSCLC cell lines, we showed that the expression of *LINC00265* is upregulated in lung cancer cells. Functional analyses indicated that knockdown of *LINC00265* reduces tumor development and prevents NSCLC cell migration and invasion. Interestingly, we found that *LINC00265* interacts with the transcription factor SIN3A to regulate its expression. Moreover, inhibiting the function of *LINC00265* and SIN3A induced autophagy and apoptosis of NSCLC cells. These results indicate that *LINC00265* plays an important role in LUAD development. They provide insights into the mechanism underlying *LINC00265* activity in lung tumorigenesis, and help to identify potential targets for lung cancer therapy.

## Materials and Methods

### Clinical tissue samples

Human lung cancer tissues and paired normal tissues were collected at the Department of Thoracic Surgery of the Affiliated Hospital of Guangdong Medical University (China). Primary tumor sites and the corresponding normal tissues were dissected and subjected to RNA extraction. All participants provided written informed consent. This research was approved by the Ethical Committee of the Affiliated Hospital of Guangdong Medical University (Approval Number: YS2022083).

### Cell lines

Human lung cell lines with different metastatic potentials (H1299, PC9, H838, A549, H1650, and H1975) and normal human bronchial epithelial (HBE) cells were purchased from Kobio Biology (Nanjing, China). H1299, PC9, H838, H1650, H1975 and HBE cells were maintained in RPMI 1640 supplemented with 10% fetal bovine serum (Gibco) and 1% penicillin-streptomycin in a humidified incubator with 5% CO_2_ at 37°C. For A549 cells, RPMI 1640 was replaced by DMEM (Gibco).

### Knockdown experiments

Small interfering RNAs (siRNAs) and small hairpin RNAs (shRNAs) were provided by Genema (Suzhou, China). The targeting sequences are listed in Suppl. Table 1. Cell transfection was performed using the Lipofectamine^®^ RNAiMAX reagent (Invitrogen, Carlsbad, CA, USA).

### RNA extraction and RT-qPCR

Total RNAs were prepared from tissues and cells using the Trizol reagent (TaKaRa, Japan). Cytoplasmic and nuclear RNAs from lung cancer cell lines were isolated using the RNA Subcellular Isolation kit (Active Motif, Shanghai, China). After reverse transcription using the PrimeScript RT reagent kit (TaKaRa, Japan), qPCR was performed using the Roche LightCycler^®^ 480 System and TB Green Premix Ex Taq II kit (TakaRa, Japan). Primer sequences for qPCR are listed in Suppl. Table 2. The 2^-ΔΔCt^ method was used to estimate relative expression levels, with GAPDH as a loading control.

### Western blot

Proteins were extracted using RIPA buffer containing protease inhibitors and quantified by bicinchoninic acid (BCA) analysis (Beyotime, China). They were separated by 10% SDS-PAGE and transferred onto polyvinylidene difluoride (PVDF) membranes (Millipore, USA). Non-specific antibody binding was blocked with 5% skimmed milk, and the membranes were incubated with primary antibodies (Suppl. Table 3) at 4°C overnight, followed by incubation with peroxidase (HRP)-conjugated secondary antibody. After several washes, protein bands were visualized using Tanon 5200 chemiluminescence imaging system (Shanghai, China) and analyzed using ImageJ Software.

### Cell proliferation and transwell assays

For proliferation assay, cells were seeded in 96-well plates with each well containing 1000 cells in 100 μL of culture medium and cultured at 37°C for an appropriate period. After adding 10 μL of (water soluble tetrazolium salt-1) WST-1 solution (Beyotime Biotechnology, Shanghai, China) to each well, the plate was further incubated for 1 h in the dark. The optical density was measured at 450 and 630 nm with a microplate reader. Transwell migration and invasion assays were performed as described previously [5].

### Colony formation assay

Cells were seeded into a 6-well plate at a density of 200 cells per well and cultured for 10 to 14 days. Colonies were washed twice with PBS and fixed with methanol for 15 min. They were stained with 0.1% crystal violet for 20 min and imaged for statistical analysis.

### Apoptosis assay

Cells were collected by treatment with free Ethylenediaminetetraacetic acid (EDTA-free) trypsin and resuspended in 100 μL of 1 × binding buffer. After staining with 5 μL Annexin V-FITC and 5 μL propidium iodide (PI)(BD Biosciences, USA) for 15 min in the dark, cells were sorted by flow cytometry and analyzed using the BD FACSDiva6.1 software (BD Biosciences).

### Tumor xenografts

Five-week-old female athymic BALB/c nude mice were maintained under specific pathogen free (SPF) conditions at the animal care facility of the Experimental Animal Center of Guangxi University of Traditional Chinese Medicine. This animal experiment was approved by the Ethics Committee of Affiliated Hospital of Guangdong Medical University (Approval Number: GDY2202217). For xenograft models, H1299 cells were stably transfected with sh*LINC00265*, or with a negative control. About 2 × 106 cells in 200 μL of sterile PBS were injected subcutaneously into the axilla of each animal. Xenografts were measured by a digital caliper every 3 days and their volumes were calculated using the following equation: volume = (length × width^2^)/2.

### RNA immunoprecipitation (RIP) assay

The interaction between *LINC00265* and SIN3A was analyzed using the EZ-Magna RIP™ RNA-Binding Protein Immunoprecipitation kit (Millipore, USA) and an antibody against SIN3A. The specificity was assayed using anti-SNRNP70 antibody and control IgG (Millipore, USA). Immunoprecipitated RNAs were reverse transcribed and analyzed by qPCR.

### Detection of autophagy flux

This was performed using a tandem fluorescent-tagged LC3 construct that produces GFP-LC3 and RFP-LC3 punctae [22]. H1299 and A549 cells were first transfected with siRNAs using the Lipofectamine^®^ RNAiMAX Reagent and cultured for 48 h. They were then washed with phosphate-buffered saline (PBS) and transfected with the RFP-GFP-LC3B plasmid (Invitrogen, Carlsbad, CA, USA). The formation of fluorescent punctae was visualized by fluorescence microscopy.

### Statistical analysis

Data were analyzed using GraphPad Prism 8.0 and presented as means ± standard deviation (SD). Student’s *t*-test was used for statistical analyses, and *p* < 0.05 was considered statistically significant. All experiments were repeated at least three times.

## Results

### Upregulation of LINC00265 predicts poor prognosis in human LUAD

**Figure 1:**
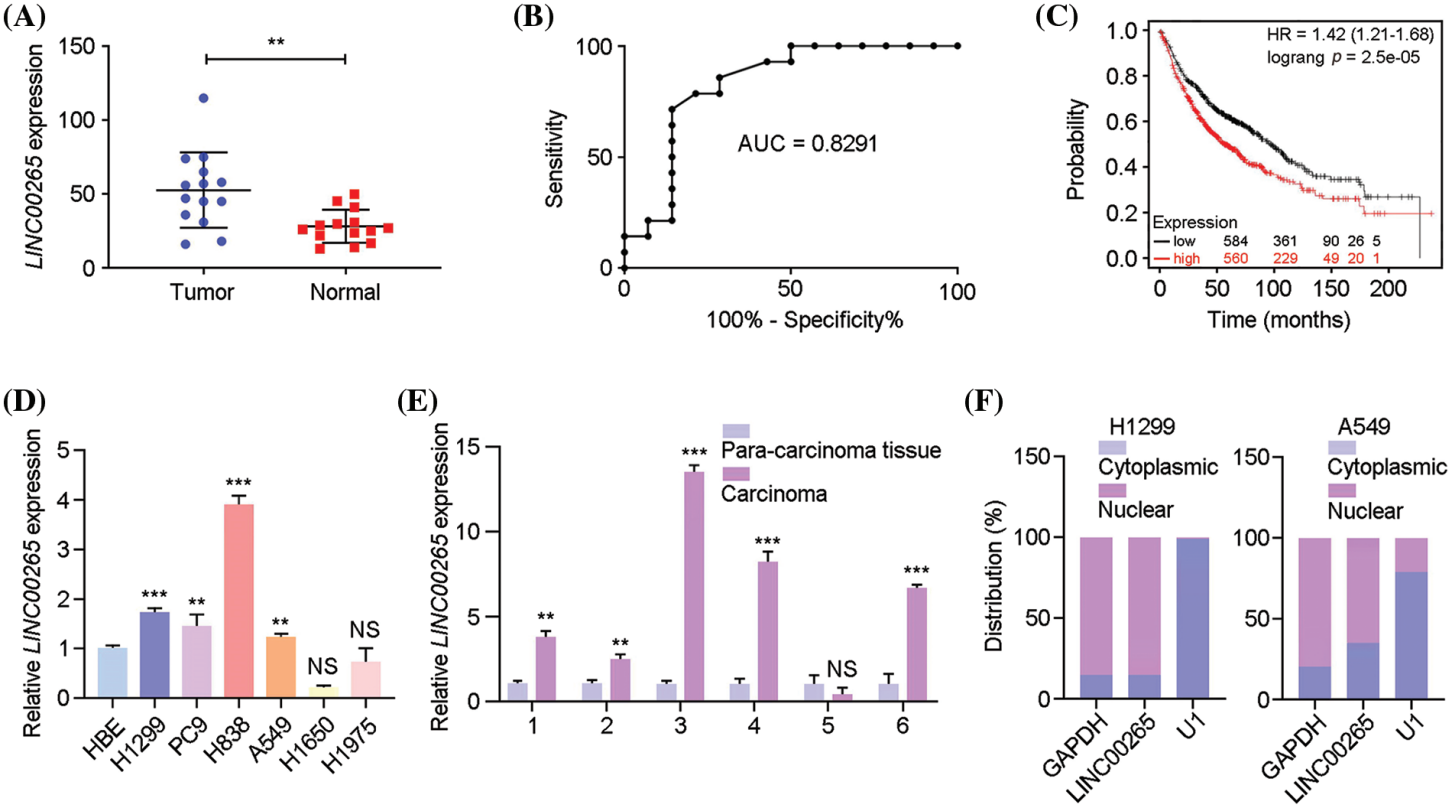
Upregulation of *LINC00265* in lung cancers and cell lines. (A) Comparison of the expression of *LINC00265* between LUAD and noncancerous lung tissues (*p* < 0.05). (B) ROC curve analysis shows the separability of *LINC00265* expression between normal and tumor tissues (AUC = 0.8291). (C) Kaplan-Meier survival analysis shows significant association of *LINC00265* expression levels with overall survival rates. (D) RT-qPCR analysis of *LIN*C00265 expression in different NSCLC cell lines. The expression of *LINC00265* in HBE cells was normalized to GAPDH and used as a reference. Data were obtained from three independent experiments (**p < 0.01; ***p < 0.001; NS, not significant). (E) The relative expression of *LINC00265* was determined in 6 pairs of LUAD tissues and para-cancer tissues by qRT-PCR (**p < 0.01; ***p < 0.001; NS, not significant). (F) Analysis of cytoplasmic and nuclear accumulation of *LINC00265* in H1299 and A549 cells.

To identify lncRNAs that may be potentially implicated in lung cancer progression, we detected lncRNA expression profifiles using available RNA-seq. The analysis indicated that *LINC00265* was significantly upregulated in lung cancer tissues, compared with adjacent tissues (Fig. 1A). This observation is consistent with the clinical information from The Cancer Genome Atlas (TCGA) database. Accordingly, analysis of ROC (receiver operating characteristic) curve showed a high degree of separability in *LINC00265* expression between tumor and normal tissues, with AUC (area under the ROC curve) higher than 0.82 (Fig. 1B). Furthermore, Kaplan-Meier analysis indicated that lung cancer patients with high levels of *LINC00265* expression had shorter overall survival (OS), suggesting a poor prognosis (Fig. 1C). We next examined *LINC00265* expression in different NSCLC cell lines and in lung cancer tissue by RT-qPCR analysis. Compared with transformed normal bronchial epithelial cells (HBE), the expression of *LINC00265* was significantly upregulated in the H1299, PC9, H838, and A549 lines, but without changes in H1650 and H1975 (Fig. 1D). The increased expression of *LINC00265* in lung cancers was further confirmed using different clinical samples, as shown by analyzing 6 paired lung cancer tissues and adjacent normal tissues (Fig. 1E). Additionally, analysis of *LINC00265* subcellular distribution using H1299 and A549 cells indicated a predominant presence in the cytoplasm, suggesting that it should mainly function in this cellular compartment (Fig. 1F). Taken together, these results suggest that *LINC00265* may play a role in the tumorigenesis of lung cancer and may represent a novel prognostic biomarker.

### Inhibition of LINC00265 reduces cell proliferation and tumor growth

**Figure 2:**
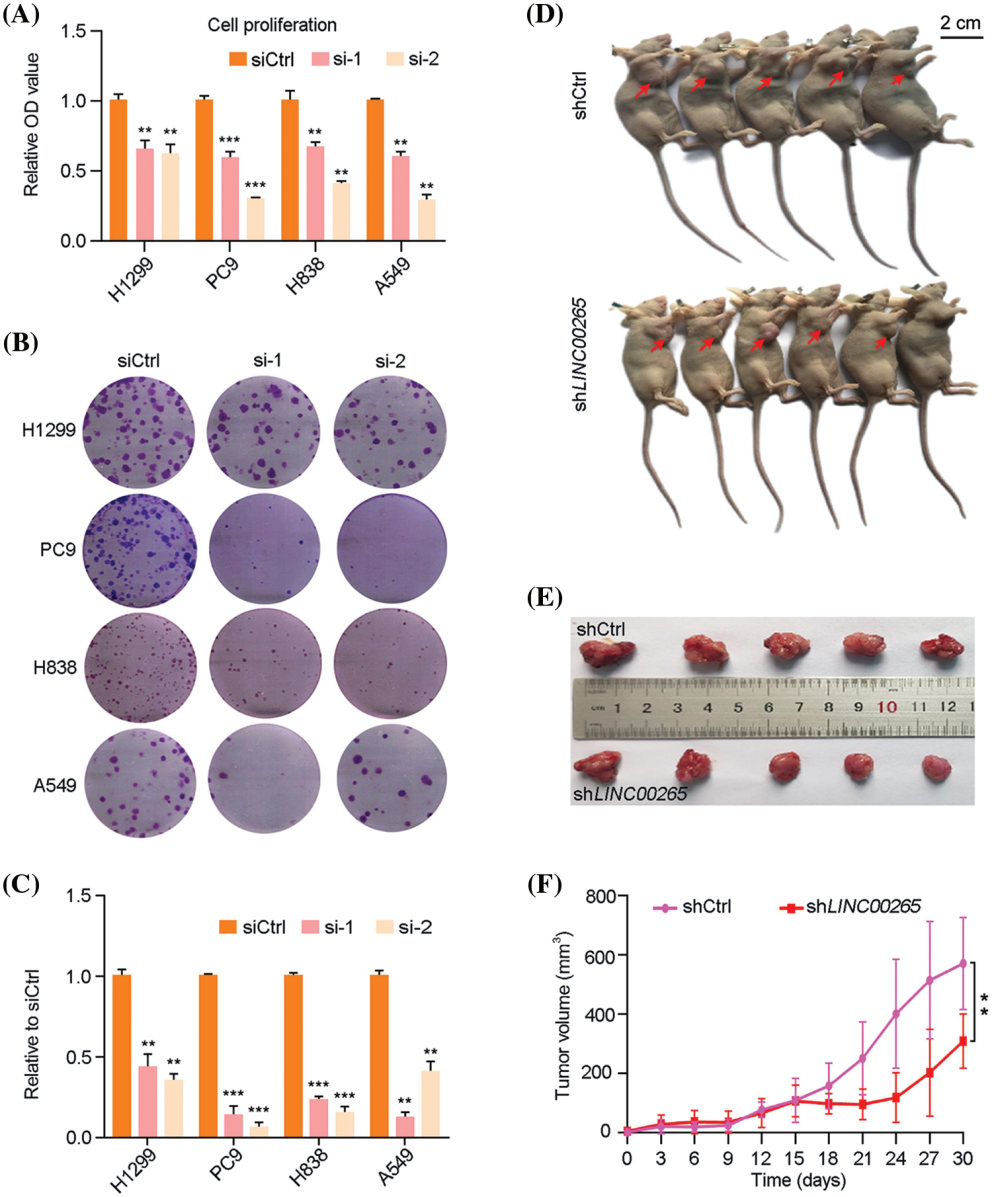
Knockdown of *LINC00265* inhibits NSCLC cell proliferation and tumor growth. (A) WST assay shows reduced proliferation of H1299, PC9, H838 and A549 cells following *LINC00265* knockdown. For each cell line, the value in siCtrl-transfected conditions is set to 1 as a reference (**p < 0.01; ***p < 0.001). (B, C) Reduced colony formation following *LINC00265* knockdown in indicated NSCLC cell lines. (D, E) Representative images shows that knockdown of *LINC00265* reduces tumor growth in xenografts. Images were taken at day 30 after implantation. (F) Statistical analysis of tumor growth overtime. Data were obtained using 5 animals for each time point (**p < 0.01).

The activity of *LINC00265* in LUAD is poorly understood. We first examined the effects of its knockdown in NSCLC cell lines by small interfering RNA (siRNA) approach. Plasmids harbor specific siRNAs targeting *LINC00265* (si*LINC00265*-1 and si*LINC00265*-2) were transfected into H1299, PC9, H838, and A549 cells. RT-qPCR analysis showed that both *LINC00265*-specific siRNAs significantly decreased the expression of *LINC00265* in different NSCLC cell lines, when compared with a siRNA (siCtrl) that does not target any sequence (Suppl. Fig. 1). Both WST and colony formation assays indicated that *LINC00265* knockdown significantly inhibited cell proliferation (Figs. 2A–2C). The tumor-inhibiting effects of *LINC00265* knockdown were further confirmed using a subcutaneous xenograft model. For this purpose, we designed a short hairpin RNA (shRNA) targeting *LINC00265* (sh*LINC00265*) and a control shRNA (shCtrl). H1299 cells were infected with slow viruses to stably express the shRNAs. We found that the multiplicity of infection at 100 particles per cells was most efficient to inhibit *LINC00265* expression and cell proliferation (Suppl. Fig. 2). Following implantation of H1299 cells expressing shCtrl or sh*LINC00265* into nude mice, tumor size in the xenografts was examined every 3 days to monitor tumor size. Consistent with the observation in NSCLC cell lines, knockdown of *LINC00265* significantly reduced tumor growth from day 18 onwards (Figs. 2D–2F). In addition, the inhibition of tumor growth is correlated with a decreased *LINC00265* expression level and reduced cell proliferation, as determined by immunocytochemical staining of the cell proliferation antigen Ki-67, autophagy associated indicators and apoptosis-related targets (Suppl. Fig. 3). These results indicate that inhibition of *LINC00265* function can efficiently prevent the progression of lung cancers.

### Knockdown of LINC00265 inhibits migration and invasion of NSCLC cells

**Figure 3:**
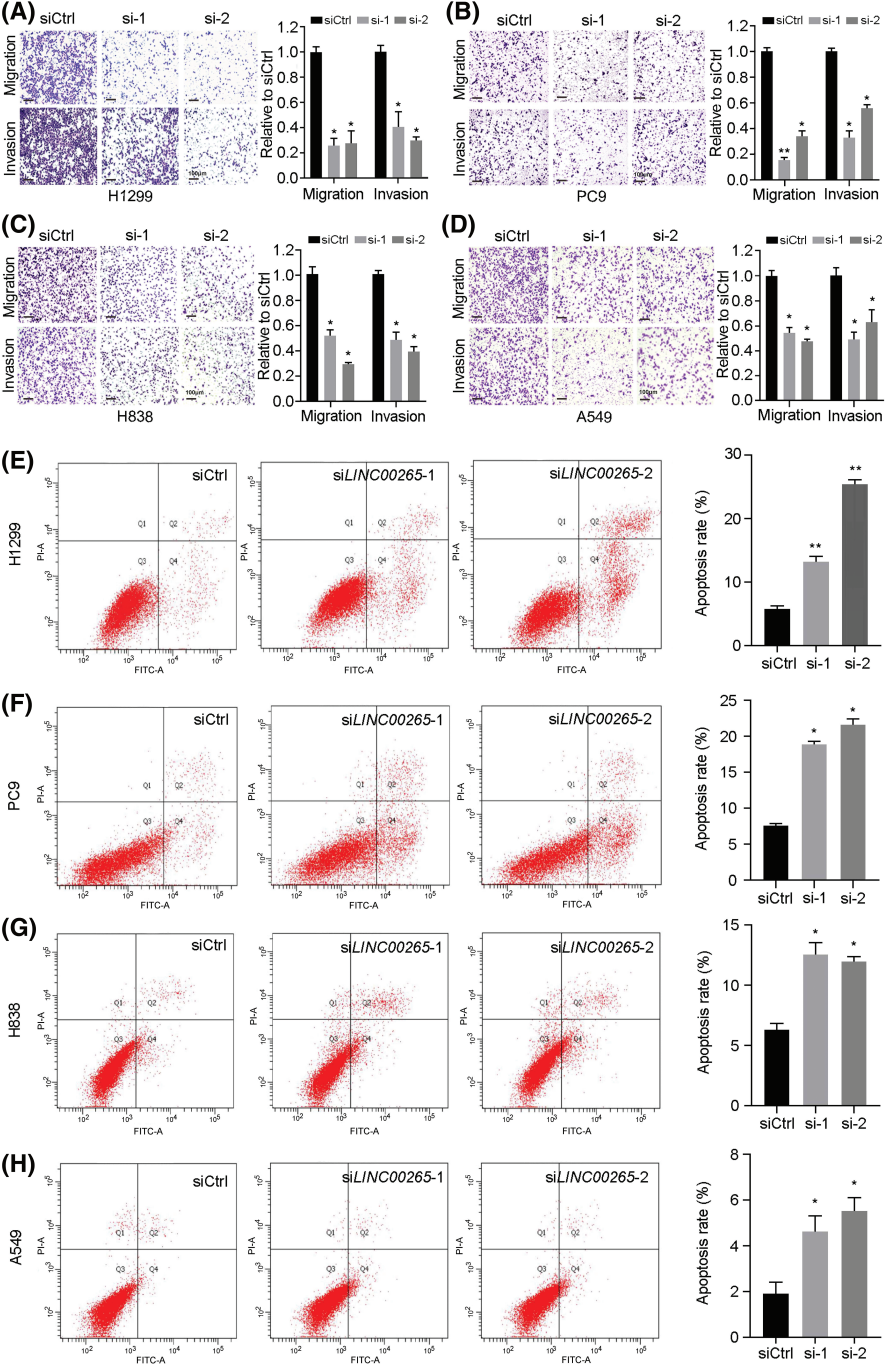
Silencing of *LINC00265* inhibits migration and invasion of NSCLC cells and induces apoptosis. (A–D) Transwell assays show the effects of *LINC00265* knockdown on NSCLC migration and invasion. The value in siCtrl-treated conditions is set to 1 as a reference. Data are the mean ± s.e.m. from three independent experiments (*p < 0.05; **p < 0.01). Scale bar, 100 μm. (E–H) Flow cytometry analyses show that knockdown of *LINC00265* increases apoptosis rate in NSCLC cells (*p < 0.05; **p < 0.01).

Transwell migration and invasion assays were used to evaluate how cell migration and invasion were affected by *LINC00265*-knockdown in NSCLC cell lines. We found that they were significantly decreased after *LINC00265* knockdown in H1299, PC9, H838, and A549 cells (Figs. 3A–3D). This suggests that *LINC00265* plays a role in the migration and invasion of NSCLC cells and that it may function as an oncogene to promote tumorigenesis or cancer progression. We then used flow cytometry to evaluate how *LINC00265* knockdown affects cell viability. The results showed that inhibition of *LINC00265* function led to increased apoptosis in different NSCLC cell lines (Figs. 3E–3H). Collectively, our functional analyses revealed an important role of *LINC00265* in the development of lung cancer.

### Induction of autophagy in NSCLC cell lines following LINC00265-knockdown

Since there exist potential pathways linking apoptosis and autophagy in cancer [23], we examined how *LINC00265*-knockdown affects autophagy in NSCLC cell lines. The expression of p62 is constantly degraded by non-selective autophagy during the formation of autophagosomes and the maturation of autophagolysosomes [24]. Its increased level results in autophagy defects, while its decreased expression promotes autophagy [25]. During the initiation of autophagy, LC3B is recruited to autophagosomal membranes and can be used as a protein marker for the formation of autophagosomes [26]. Beclin-1 is also involved in autophagosome formation and functions to inhibit tumor growth [6]. The expression of other proteins, such as mTOR, P70 and AMPK (AMP-activated protein kinase), is also associated with autophagy in cell survival and cell death [27].

**Figure 4:**
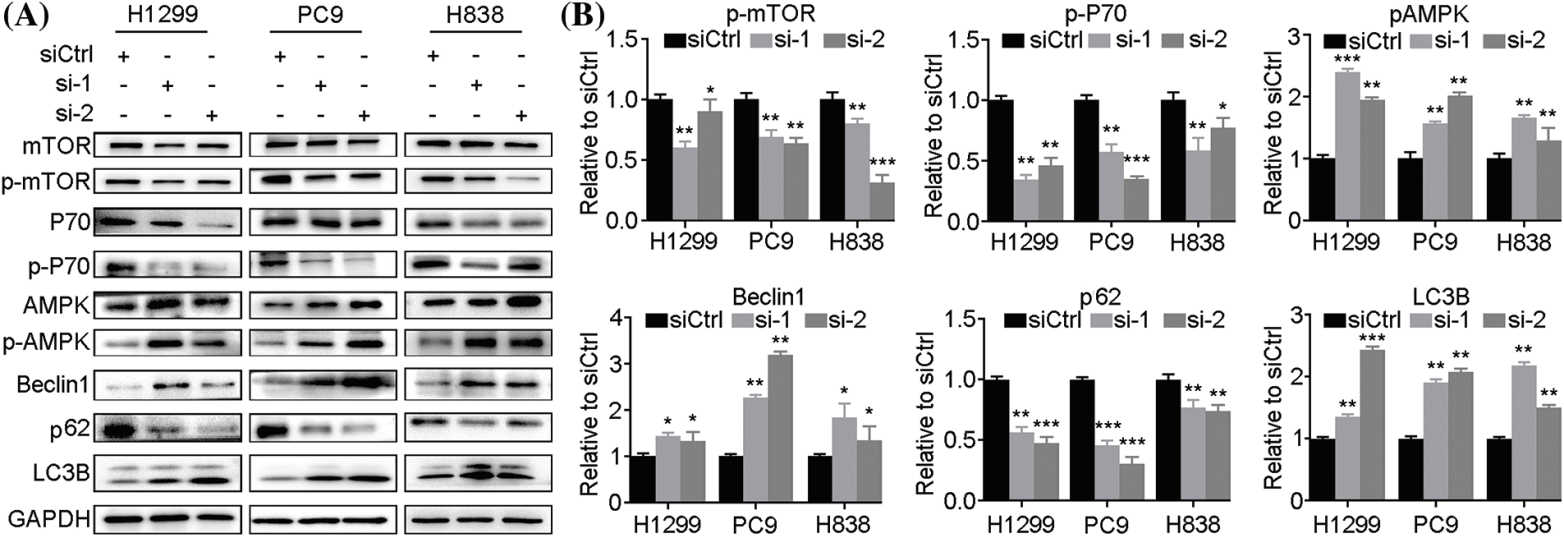
Knockdown of *LINC00265* induces autophagy. (A) Western blot analyses of autophagy-related proteins following *LINC00265* knockdown in NSCLC cell lines. (B) Quantification of western blots shows the decreased expression of p-mTOR, p-P70 and p62, but increased expression of p-AMPK, Beclin1 and LC3B, indicating autophagy activation. The value in siCtrl-treated conditions is set to 1 as a reference (*p < 0.05; **p < 0.01; ***p < 0.001).

**Figure 5:**
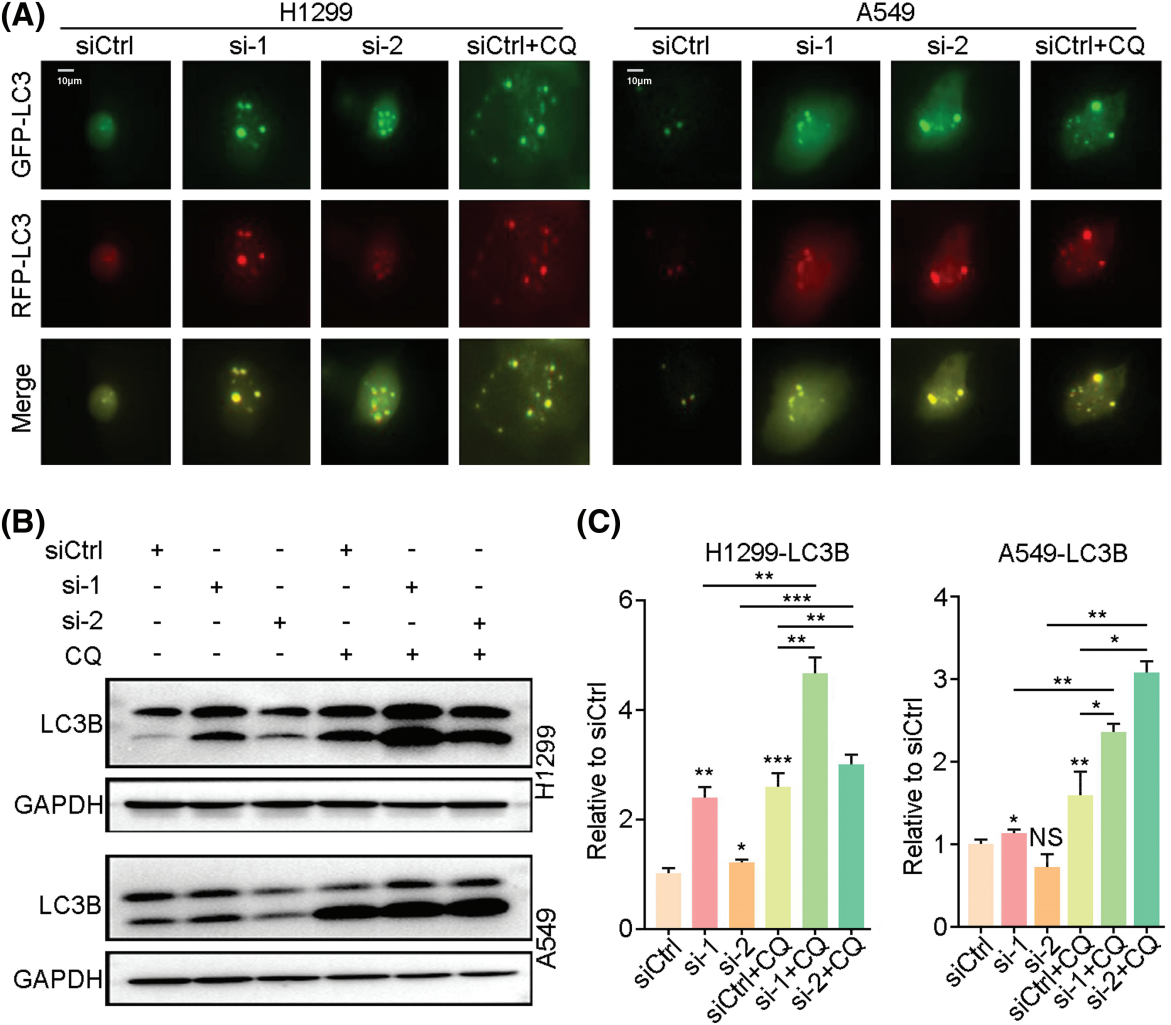
Knockdown of *LINC00265* enhances autophagy flux. (A) Representative images show increased formation of autophagosomes after knockdown of *LINC00265* in H1299 and A549 cells. Scale bar, 10 μm. (B) Western blot analyses of LC3B expression following knockdown of *LINC00265* in H1299 and A549 cells treated with CQ. (C) Quantification of western blots. Increased LC3B expression in CQ-treated conditions suggests enhanced autophagy flux (*p < 0.05; **p < 0.01; ***p < 0.001; NS, not significant).

By western blot analysis of autophagy-related signature proteins, we found that expression levels of mTOR, p-mTOR, P70, p-P70 and p62 were decreased in H1299, PC9 and H838 cells at 72h after *LINC00265*-knockdown, concomitantly with increased expression levels of AMPK, p-AMPK, Beclin1 and LC3B (Fig. 4A, Suppl. Fig. 4). In animal models, autophagy-related proteins showed similar expression as in NSCLC cell lines (Suppl. Fig. 5). These results are reminiscent of autophagy activation, suggesting that *LINC00265*-knockdown can trigger autophagy in lung cancer cells. Furthermore, we examined the effects of *LINC00265*-knockdown on autophagy flux by fluorescence microscopy to monitor how this induces the formation of autolysosomes. H1299 and A549 cells were infected with a lentiviral vector harboring a tandem fluorescent-tagged LC3 to monitor the formation of GFP-LC3 and RFP-LC3 punctae. Since the GFP signal is quenched in acidic lysosomes, RFP punctae will be displayed in cells containing autolysosomes, which indicate autophagy termination. There were very few red and orange punctae in uninfected H1299 and A549 cells. However, silencing of *LINC00265* significantly increased the number of red and orange punctae (Fig. 5A). To further confirm the induction of autophagy, we treated LINC00265-knockdwon cells with chloroquine (CQ), a known inhibitor of autophagy, because it can prevent the fusion of autophagosomes with lysosomes [28,29]. Therefore, a further increase in LC3B protein level is correlated with enhanced autophagy flux [30]. As expected, western blot analysis showed that knockdown of *LINC00265* in H1299 and A549 cells followed by treatment with CQ (10 µM) further increased LC3B expression (Figs. 5B, 5C). Thus, we can conclude that inhibiting the function of *LINC00265* leads to activation of autophagy in NSCLC cells.

### LINC00265 interacts with SIN3A protein and regulates its stability

**Figure 6:**
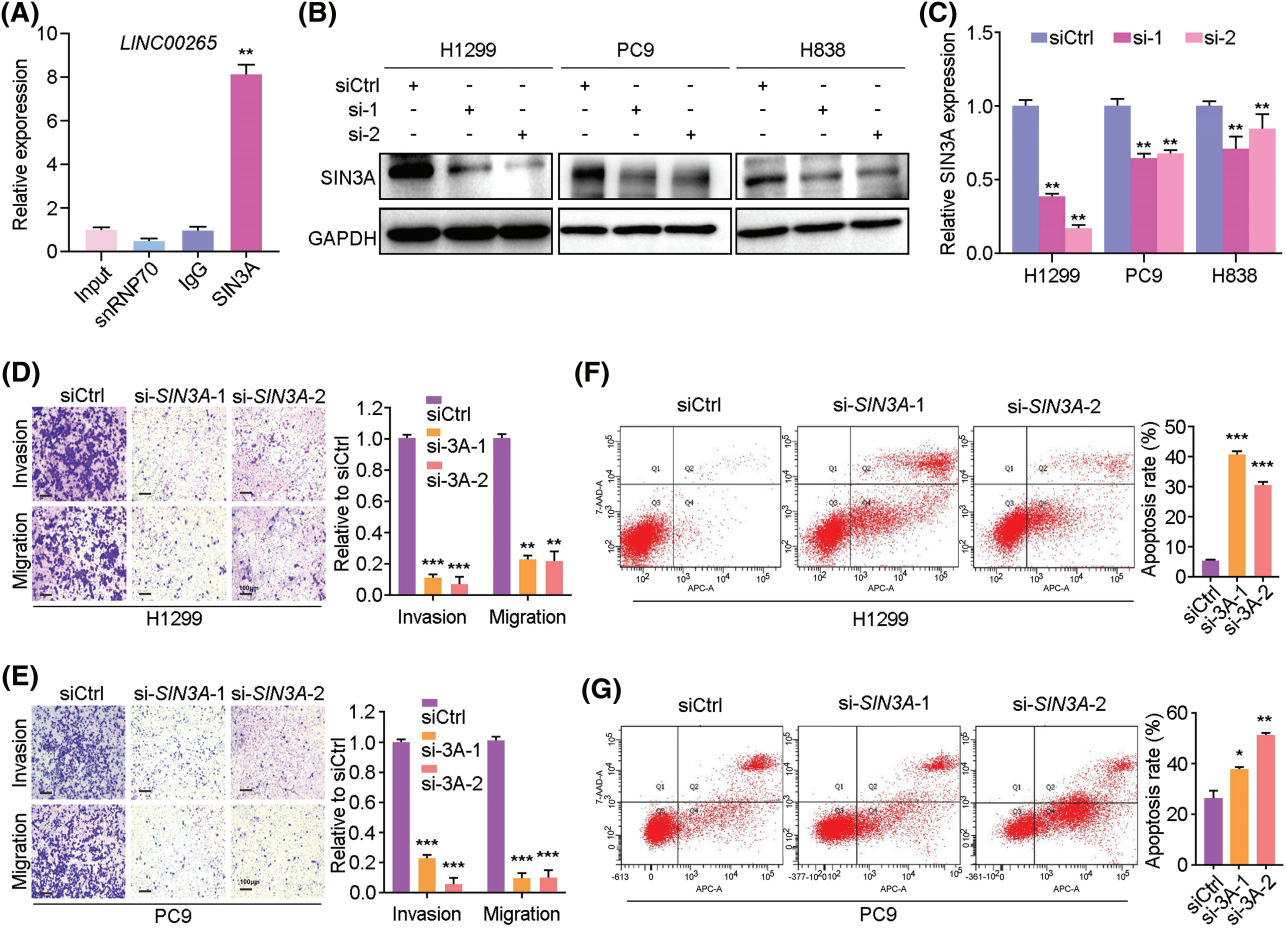
Interaction between *LINC00265* and SIN3A protein. (A) RIP-qPCR assay shows the interaction between *LINC00265* and SIN3A protein (**p < 0.01). Samples were subjected to RNA purification using anti-SIN3A antibody, anti-SNRNP70 or control IgG. The value from input sample is set to 1. (B, C) Western blot and quantification show reduced SIN3A protein levels after knockdown of *LINC00265* in NSCLC cells (**p < 0.01). (D, E) Transwell assays show that knockdown of SIN3A reduces migration and invasion of H1299 and PC9 cells. Data are expressed as the mean ± s.e.m. from three independent experiments (**p < 0.01; ***p < 0.001). Scale bar, 100 μm. (F, G) Flow cytometry analysis shows that knockdown of SIN3A increases apoptosis rate in NSCLC cells. Data are the mean ± s.e.m. from three independent experiments (*p < 0.05; **p < 0.01; ***p < 0.001).

To gain further insight into the mechanism underlying *LINC00265* in LUAD, we used bioinformatics tools (GeneCards) to predict its interacting proteins. Among different possible candidates, the transcriptional co-repressor SIN3A protein was particularly interesting because its implication in tumorigenesis [21,31,32]. We further verified the physical interaction between *LINC00265* and SIN3A by RIP-qPCR. The results clearly showed that *LINC00265* transcripts were significantly enriched by SIN3A-specific antibody (Fig. 6A), suggesting complex formation between *LINC00265* and SIN3A. To determine the biological relevance of this interaction, we analyzed SIN3A protein expression by western blot and found a decrease in *LINC00265*-knockdown cells (Figs. 6B, 6C). In light of the binding of *LINC00265* with SIN3A protein, these results indicate that it may function in regulating the stability of SIN3A in NSCLC cells.

We then examined the function of SIN3A in cell proliferation using two different siRNAs. The knockdown efficiency was verified by qRT-PCR in H1299, PC9, H838 and A549 cells, as well as by western blotting and WST assay in H1299 and PC9 cells (Suppl. Fig. 6). Analysis by transwell assays indicated that silencing of SIN3A in NSCLC cells strongly reduced cell invasion and migration, when compared with the control siRNA (Figs. 6D, 6E). This was correlated with an increased apoptosis, as determined by cytometry analysis (Figs. 6F, 6G).

### Knockdown of SIN3A induces autophagy in NSCLC cells

**Figure 7:**
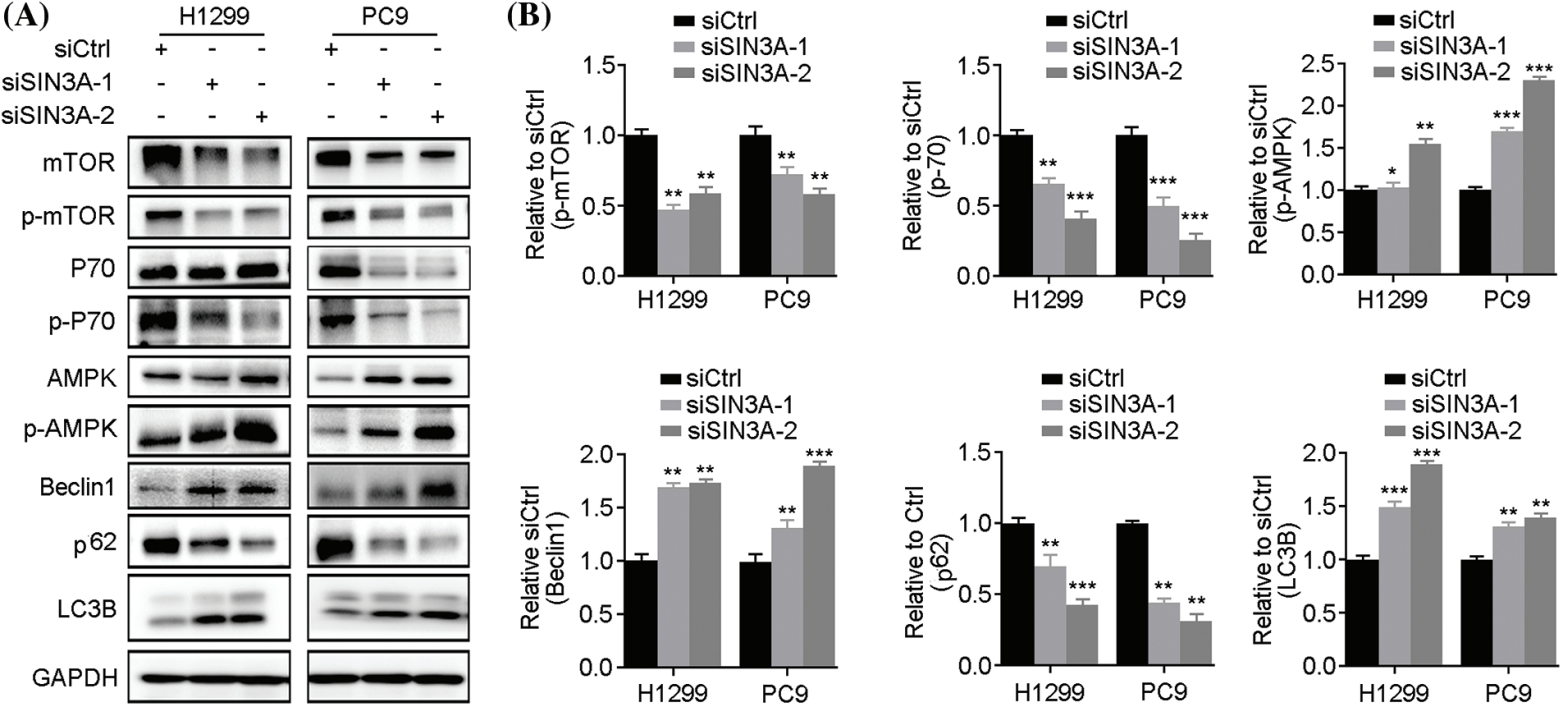
Knockdown of SIN3A induces autophagy. (A) Western blot analyses show the expression of autophagy-related proteins following SIN3A knockdown in NSCLC cell lines. (B) Quantification of western blots shows the decreased expression of p-mTOR, p-P70 and p62, but increased expression of p-AMPK, Beclin1 and LC3B, indicating autophagy activation (*p < 0.05; **p < 0.01; ***p < 0.001).

Since *LINC00265* regulates autophagy, its interaction with SIN3A raises the possibility that the latter may have a similar function. We thus examined the effects of SIN3A knockdown on autophagy by western blot. The results showed that protein levels of p-AMPK, Beclin1 and LC3B were increased, whereas the expression of p-mTOR, p-P70 and p62 was reduced in H1299 and PC9 cells (Fig. 7). Therefore, this expression profile of autophagy-related proteins is similar as observed in *LINC00265*-knockdown cells, suggesting that *LINC00265* and SIN3A may function together to regulate lung cancer development. Altogether, these observations identify *LINC00265* as an interaction partner of SIN3A in LUAD. It is possible that *LINC00265* stabilizes SIN3A protein to promote its oncogenic activity. This is consistent with its implication in tumorigenesis [21].

## Discussion

LncRNAs not only function in cancer immunity, metabolism and metastasis, but also play an important role in drug resistance [33–36]. Dysregulation of lncRNAs have been observed in various diseases and are critically implicated in cancer development [37,38]. *LINC00265* displays oncogenic activity in several cancers, including hepatocellular carcinoma, bladder cancer, and gastric and colorectal cancers [11,39–41]. However, how *LINC00265* functions in lung cancer remains elusive. In this study, we found that *LINC00265* is significantly upregulated in lung cancer, which is associated with poor patient survival. Moreover, we showed that knockdown of *LINC00265* reduces cancer cell proliferation and tumor growth by inducing autophagy and apoptosis. Molecularly, *LINC00265* interacts with the oncogenic protein SIN3A and regulates its stability. These findings provide insights into the regulatory role of *LINC00265* in LUAD progression. Thus, targeting *LINC00265* could be a potential strategy for treatment of lung cancer.

It is well established that cancer cells display cuproptosis-related ferroptosis and apoptosis-linked autophagy-dependent cell death [42,43]. Silencing of *LINC00265* could enhance autophagy flux because there was a further increased protein level of LC3B in the presence chloroquine. This suggests that knockdown of *LINC00265* triggers autophagy and apoptosis, thereby preventing LUAD progression. On the contrary, an increased expression of *LINC00265* should prevent autophagy-dependent cell death, thus promoting LUAD proliferation. Our results imply that *LINC00265* should function to promote LUAD development by inhibiting autophagy-dependent cell death. There is evidence that autophagy displays stage-dependent functions in tumorigenesis. It acts as a tumor suppressor to inhibit the expression of oncogenic genes at initial stages but as a pro-tumor factor by promoting tumor cell survival at advanced stages [44]. Although the relationship between *LINC00265*-regulated autophagy and apoptosis remains unclear and needs further investigations, there is evidence suggesting that common pathways can regulate these processes [43]. Our results indicate that *LINC00265* may function in both processes. Future works will be necessary how *LINC00265* regulates autophagy-dependent or independent cell death.

SIN3A is a member of the SIN3 family and functions in the HDAC complex. It plays essential roles in various cellular processes linked to cancer pathogenesis and progression [18]. Nevertheless, how it functions in tumor biology is still subject of debate [16]. There is a possibility that SIN3A displays oncogenic or anti-tumor activity dependent on its biochemical interaction with specific protein partners, genetic interaction with other genes or regulation of target genes [17,20]. It has been shown that SIN3A could function as a tumor suppressor by regulating gene expression involved in cell invasion [17,19,20]. More recent works indicated that it interacts with and contributes to the oncogenic potential of the transcription factor STAT3 by repressing the transcription of tumor suppressor genes. Thus, interfering with its expression causes transcriptional activation tumor suppressor genes and reduces tumorigenic potential of anaplastic large-cell lymphoma by increasing tumor cell death [21]. Our present observations further support an oncogenic activity of SIN3A. They suggest that SIN3A may be implicated in regulating autophagy in NSCLC cells.

We found that *LINC00265* binds to and stabilizes SIN3A protein in lung cancer cells, thus identifying a novel partner for this LncRNA. Silencing of *LINC00265* results in a decreased expression of SIN3A expression. Of note, inhibiting the function of SIN3A produced similar effects as *LINC00265* knockdown in NSCLC cell lines, suggesting they may function together in LUAD progression. Since *LINC00265* interacts with SIN3A, its oncogenic activity may be mediated by SIN3A. There is a possibility that knockdown of SIN3A inhibits NSCLC migration and invasion at least partially by activating autophagy. Thus, our results support a pro-tumor activity of this epigenetic regulator. Consistent with this conclusion, it has been shown that the SIN3A promotes cell survival in different cancers by forming a complex with STAT3 to silence tumor repressor genes [21]. Interestingly, *LINC00265* has been also shown to promote metastasis of acute lymphoblastic leukemia by regulating STAT3 signaling [14]. This raises a possibility that *LINC00265* cooperates with SIN3A to enhance STAT3 signaling in LUAD. At present, it is still unclear how *LINC00265* regulates the stability of SIN3A protein. However, *LINC00265* has been shown to promote colorectal tumorigenesis through activation of Wnt/ß-catenin signaling. Mechanistically, *LINC00265* increases the expression of ZMIZ2, which in turn recruits the deubiquitylase USP7 to stabilize β-catenin protein. Whether *LINC00265* stabilizes SIN3A protein through a similar mechanism merits further investigation.

## Conclusions

In summary, this study shows that upregulation of *LINC00265* in LUAD leads to reduced overall survival rate in patients and that inhibition of *LINC00265* can prevent tumor progression. Our results also indicate that *LINC00265* regulates autophagy and apoptosis likely through interaction with SIN3A. These findings provide insights into the mechanism underlying *LINC00265* activity in lung tumorigenesis.

## Supplementary Materials

Figure S1. Assay of LINC00265 knockdown efficiency using siRNAs. RT-qPCR analysis of LINC00265 expression in NSCLC cells lines. The value in siCtrl-treated conditions is used as a reference (*, p < 0.05; **, p < 0.01).

Figure S2. Assay of LINC00265 knockdown efficiency using shRNAs. (A) RT-qPCR analysis of LINC00265 expression in H1299 cells. (B) WST assay shows efficient inhibition of cell proliferation after knockdown by MOI-100. (*, p < 0.05; **, p < 0.01; ***, p < 0.001).

Figure S3. Knockdown of LINC00265 inhibits lung cancer growth and induces autophagy in vivo. Ki-67, LC3B, p62, PARP1 and SIN3A expression in xenografs was examined by immunohistochemistry staining. Scale bar, 100µm.

Figure S4. Quantification of autophagy-related proteins following LINC00265 knockdown. The data correspond to western blots shown in Figure 4. The value in siCtrl-treated cells is set to 1 as a reference (*, p < 0.05; **, p < 0.01; ***, p < 0.001).

Figure S5. Knockdown of LINC00265 induces autophagy in xenograft tumors. (A) Western blot analyses show that knockdown of LINC00265 activates autophagy and reduces SIN3A expression in xenograft tumors. (B) Quantification of western blots compares the expression levels of indicated proteins. (*, p < 0.05; **, p < 0.01; ***, p < 0.001).

Figure S6. Assay of SIN3A knockdown efficiency. (A) RT-qPCR analysis of SIN3A expression in NSCLC cell lines. (B) Western blot analysis of SIN3A expression in H1299 and PC9 cells. (C) WST assay shows inhibition of cell proliferation after knockdown of SIN3A. (*, p < 0.05; **, p < 0.01; ***, p < 0.001).

## Data Availability

All data that support the findings of this study are available from the corresponding authors upon reasonable request.

## Supplementary Materials

The supplementary material is available online at https://doi.org/10.32604/or.2023.030771.

## References

[1] Testa, U., Castelli, G., Pelosi, E. (2018). Lung cancers: Molecular characterization, clonal heterogeneity and evolution, and cancer stem cells. Cancers*,* 10 *(*8*),* 248. 10.3390/cancers10080248; 30060526 PMC6116004

[2] Sung, H., Ferlay, J., Siegel, R. L., Laversanne, M., Soerjomataram, I. et al. (2021). Global cancer statistics 2020: Globocan estimates of incidence and mortality worldwide for 36 cancers in 185 countries. CA: A Cancer Journal for Clinicians*,* 71 *(*3*),* 209–249; 33538338 10.3322/caac.21660

[3] Bade, B. C., Dela Cruz, C. S. (2020). Lung cancer 2020: Epidemiology, etiology, and prevention. Clinics in Chest Medicine*,* 41 *(*1*),* 1–24. 10.1016/j.ccm.2019.10.001; 32008623

[4] Ginn, L., Shi, L., Montagna, M., Garofalo, M. (2020). Lncrnas in non-small-cell lung cancer. Non-Coding RNA*,* 6 *(*3*),* 25. 10.3390/ncrna6030025; 32629922 PMC7549371

[5] Su, W., Feng, S., Chen, X., Yang, X., Mao, R. et al. (2018). Silencing of long noncoding RNA MIR22HG triggers cell survival/death signaling via oncogenes YBX1, MET, and p21 in lung cancer. Cancer Research*,* 78 *(*12*),* 3207–3219. 10.1158/0008-5472.CAN-18-0222; 29669758 PMC6004254

[6] Liang, X. H., Jackson, S., Seaman, M., Brown, K., Kempkes, B. et al. (1999). Induction of autophagy and inhibition of tumorigenesis by beclin 1. Nature*,* 402 *(*6762*),* 672–676. 10.1038/45257; 10604474

[7] Vencken, S. F., Greene, C. M., McKiernan, P. J. (2015). Non-coding RNA as lung disease biomarkers. Thorax*,* 70 *(*5*),* 501–503. 10.1136/thoraxjnl-2014-206193; 25550385

[8] Li, D. S., Ainiwaer, J. L., Sheyhiding, I., Zhang, Z., Zhang, L. W. (2016). Identification of key long non-coding rnas as competing endogenous RNAs for mirna-mrna in lung adenocarcinoma. European Review for Medical and Pharmacological Sciences*,* 20 *(*11*),* 2285–2295; 27338053

[9] Ge, H., Yan, Y., Yue, C., Liang, C., Wu, J. (2019). Long noncoding RNA linc00265 targets EGFR and promotes deterioration of colorectal cancer: A comprehensive study based on data mining and *in vitro* validation. OncoTargets and Therapy*,* 12*,* 10681–10692. 10.2147/OTT 31824175 PMC6901053

[10] Zhu, Y., Gu, L., Lin, X., Cui, K., Liu, C. et al. (2020). Linc00265 promotes colorectal tumorigenesis via ZMIZ2 and USP7-mediated stabilization of β-catenin. Cell Death and Differentiation*,* 27 *(*4*),* 1316–1327. 10.1038/s41418-019-0417-3; 31527801 PMC7206056

[11] Sun, S., Li, W., Ma, X., Luan, H. (2020). Long noncoding RNA linc00265 promotes glycolysis and lactate production of colorectal cancer through regulating of mir-216b-5p/trim44 axis. Digestion*,* 101 *(*4*),* 391–400. 10.1159/000500195; 31079111

[12] Ma, L., Kuai, W. X., Sun, X. Z., Lu, X. C., Yuan, Y. F. (2018). Long noncoding RNA linc00265 predicts the prognosis of acute myeloid leukemia patients and functions as a promoter by activating PI3K-AKT pathway. European Review for Medical and Pharmacological Sciences*,* 22 *(*22*),* 7867–7876; 30536332 10.26355/eurrev_201811_16412

[13] Zhang, F., Li, Q., Zhu, K., Zhu, J., Li, J. et al. (2020). Lncrna linc00265/mir-485-5p/irf2-mediated autophagy suppresses apoptosis in acute myeloid leukemia cells. American Journal of Translational Research*,* 12 *(*6*),* 2451–2462; 32655783 PMC7344095

[14] Zhao, D., Xing, Q., Song, H., Zhao, Y., Guo, G. (2021). Linc00265/mir-4500 axis accelerates acute lymphoblastic leukemia progression by enhancing stat3 signals. Cancer Management and Research*,* 13*,* 8147–8156. 10.2147/CMAR.S274590; 34737643 PMC8560060

[15] Xiao, Q., Lin, C., Peng, M., Ren, J., Jing, Y. et al. (2022). Circulating plasma exosomal long non-coding RNAs linc00265, linc00467, uca1, and snhg1 as biomarkers for diagnosis and treatment monitoring of acute myeloid leukemia. Frontiers in Oncology*,* 12*,* 1033143. 10.3389/fonc.2022.1033143; 36276083 PMC9585262

[16] Kadamb, R., Mittal, S., Bansal, N., Batra, H., Saluja, D. (2013). Sin3: Insight into its transcription regulatory functions. European Journal of Cell Biology*,* 92 *(*8–9*),* 237–246; 24189169 10.1016/j.ejcb.2013.09.001

[17] Kadamb, R., Leibovitch, B. A., Farias, E. F., Dahiya, N., Suryawanshi, H. et al. (2022). Invasive phenotype in triple negative breast cancer is inhibited by blocking sin3a-PF1 interaction through KLF9 mediated repression of ITGA6 and ITGB1. Translational Oncology*,* 16*,* 101320. 10.1016/j.tranon.2021.101320; 34968869 PMC8718897

[18] Bansal, N., David, G., Farias, E., Waxman, S. (2016). Emerging roles of epigenetic regulator sin3 in cancer. Advances in Cancer Research*,* 130*,* 113–135. 10.1016/bs.acr.2016.01.006; 27037752

[19] Suzuki, H., Ouchida, M., Yamamoto, H., Yano, M., Toyooka, S. et al. (2008). Decreased expression of the sin3a gene, a candidate tumor suppressor located at the prevalent allelic loss region 15q23 in non-small cell lung cancer. Lung Cancer*,* 59 *(*1*),* 24–31. 10.1016/j.lungcan.2007.08.002; 17854949

[20] Das, T. K., Sangodkar, J., Negre, N., Narla, G., Cagan, R. L. (2013). Sin3a acts through a multi-gene module to regulate invasion in drosophila and human tumors. Oncogene*,* 32 *(*26*),* 3184–3197. 10.1038/onc.2012.326; 22890320 PMC3696049

[21] Gambi, G., di Simone, E., Basso, V., Ricci, L., Wang, R. et al. (2019). The transcriptional regulator sin3a contributes to the oncogenic potential of stat3. Cancer Research*,* 79 *(*12*),* 3076–3087. 10.1158/0008-5472.CAN-18-0359; 30692217

[22] Kimura, S., Noda, T., Yoshimori, T. (2007). Dissection of the autophagosome maturation process by a novel reporter protein, tandem fluorescent-tagged lc3. Autophagy*,* 3 *(*5*),* 452–460. 10.4161/auto.4451; 17534139

[23] Su, M., Mei, Y., Sinha, S. (2013). Role of the crosstalk between autophagy and apoptosis in cancer. Journal of Oncology*,* 2013*,* 102735; 23840208 10.1155/2013/102735 PMC3687500

[24] Moscat, J., Diaz-Meco, M. T. (2009). P62 at the crossroads of autophagy, apoptosis, and cancer. Cell*,* 137 *(*6*),* 1001–1004. 10.1016/j.cell.2009.05.023; 19524504 PMC3971861

[25] Mathew, R., Karp, C. M., Beaudoin, B., Vuong, N., Chen, G. et al. (2009). Autophagy suppresses tumorigenesis through elimination of p62. Cell*,* 137 *(*6*),* 1062–1075. 10.1016/j.cell.2009.03.048; 19524509 PMC2802318

[26] Tanida, I., Ueno, T., Kominami, E. (2008). Lc3 and autophagy. Methods in Molecular Biology*,* 445*,* 77–88. 10.1007/978-1-59745-157-4 18425443

[27] Das, G., Shravage, B. V., Baehrecke, E. H. (2012). Regulation and function of autophagy during cell survival and cell death. Cold Spring Harbor Perspectives in Biology*,* 4 *(*6*),* a008813; 22661635 10.1101/cshperspect.a008813 PMC3367545

[28] Klionsky, D. J., Abdel-Aziz, A. K., Abdelfatah, S., Abdellatif, M., Abdoli, A. et al. (2021). Guidelines for the use and interpretation of assays for monitoring autophagy. Autophagy*,* 17 *(*1*),* 1–382. 10.1080/15548627.2020.1797280; 33634751 PMC7996087

[29] Mauthe, M., Orhon, I., Rocchi, C., Zhou, X., Luhr, M. et al. (2018). Chloroquine inhibits autophagic flux by decreasing autophagosome-lysosome fusion. Autophagy*,* 14 *(*8*),* 1435–1455. 10.1080/15548627.2018.1474314; 29940786 PMC6103682

[30] Mizushima, N., Yoshimori, T. (2007). How to interpret LC3 immunoblotting. Autophagy*,* 3 *(*6*),* 542–545. 10.4161/auto.4600; 17611390

[31] Solaimani, P., Wang, F., Hankinson, O. (2014). Sin3a, generally regarded as a transcriptional repressor, is required for induction of gene transcription by the aryl hydrocarbon receptor. The Journal of Biological Chemistry*,* 289 *(*48*),* 33655–33662. 10.1074/jbc.M114.611236; 25305016 PMC4246116

[32] Lewis, M. J., Liu, J., Libby, E. F., Lee, M., Crawford, N. P. et al. (2016). Sin3a and sin3b differentially regulate breast cancer metastasis. Oncotarget*,* 7 *(*48*),* 78713–78725. 10.18632/oncotarget.v7i48 27780928 PMC5340233

[33] Jiang, M. C., Ni, J. J., Cui, W. Y., Wang, B. Y., Zhuo, W. (2019). Emerging roles of lncrna in cancer and therapeutic opportunities. American Journal of Cancer Research*,* 9 *(*7*),* 1354–1366; 31392074 PMC6682721

[34] Wu, P., Mo, Y., Peng, M., Tang, T., Zhong, Y. et al. (2020). Emerging role of tumor-related functional peptides encoded by lncrna and circrna. Molecular Cancer*,* 19 *(*1*),* 22. 10.1186/s12943-020-1147-3; 32019587 PMC6998289

[35] Lin, Y. H. (2020). Crosstalk of lncrna and cellular metabolism and their regulatory mechanism in cancer. International Journal of Molecular Sciences*,* 21 *(*8*),* 2947. 10.3390/ijms21082947; 32331347 PMC7215767

[36] Shi, Q., Li, Y., Li, S., Jin, L., Lai, H. et al. (2020). Lncrna DILA1 inhibits cyclin D1 degradation and contributes to tamoxifen resistance in breast cancer. Nature Communications*,* 11 *(*1*),* 5513. 10.1038/s41467-020-19349-w; 33139730 PMC7608661

[37] Peng, W. X., Koirala, P., Mo, Y. Y. (2017). Lncrna-mediated regulation of cell signaling in cancer. Oncogene*,* 36 *(*41*),* 5661–5667. 10.1038/onc.2017.184; 28604750 PMC6450570

[38] Munschauer, M., Nguyen, C. T., Sirokman, K., Hartigan, C. R., Hogstrom, L. et al. (2018). The norad lncrna assembles a topoisomerase complex critical for genome stability. Nature*,* 561 *(*7721*),* 132–136. 10.1038/s41586-018-0453-z; 30150775

[39] Yang, Z., OuYang, X., Zheng, L., Dai, L., Luo, W. (2021). Long intergenic noncoding RNA00265 promotes proliferation of gastric cancer via the microrna-144-3p/chromobox 4 axis. Bioengineered*,* 12 *(*1*),* 1012–1025. 10.1080/21655979.2021.1876320; 33464142 PMC8291797

[40] Zhi, Y., Sun, F., Cai, C., Li, H., Wang, K. et al. (2021). Linc00265 promotes the viability, proliferation, and migration of bladder cancer cells via the mir-4677-3p/fgf6 axis. Human & Experimental Toxicology*,* 40 *(*12_suppl*),* S434–S446. 10.1177/09603271211043479; 34591706

[41] Ge, B., Zhang, X., Zhou, W., Mo, Y., Su, Z. H. et al. (2022). Linc00265 promotes metastasis and progression of hepatocellular carcinoma by interacting with E2F1 at the promoter of CDK2. Cell Journal*,* 24 *(*6*),* 294–301; 35892231 10.22074/cellj.2022.8035 PMC9315211

[42] Wang, T., Jiang, X., Lu, Y., Ruan, Y., Wang, J. (2023). Identification and integration analysis of a novel prognostic signature associated with cuproptosis-related ferroptosis genes and relevant lncrna regulatory axis in lung adenocarcinoma. Sedentary Life and Nutrition*,* 15 *(*5*),* 1543–1563. 10.18632/aging.204561 PMC10042693 36881404

[43] Liu, G., Pei, F., Yang, F., Li, L., Amin, A. D. et al. (2017). Role of autophagy and apoptosis in non-small-cell lung cancer. International Journal of Molecular Sciences*,* 18 *(*2*),* 367. 10.3390/ijms18020367; 28208579 PMC5343902

[44] Chavez-Dominguez, R., Perez-Medina, M., Lopez-Gonzalez, J. S., Galicia-Velasco, M., Aguilar-Cazares, D. (2020). The double-edge sword of autophagy in cancer: From tumor suppression to pro-tumor activity. Frontiers in Oncology*,* 10*,* 578418. 10.3389/fonc.2020.578418; 33117715 PMC7575731

