# Correction: Silencing of the long non-coding RNA LINC00265 triggers autophagy and apoptosis in lung cancer by reducing protein stability of SIN3A oncogene

**DOI:** 10.32604/or.2024.061822

**Published:** 2025-04-18

**Authors:** XIAOBI HUANG, CHUNYUAN CHEN, YONGYANG CHEN, HONGLIAN ZHOU, YONGHUA CHEN, ZHONG HUANG, YULIU XIE, BAIYANG LIU, YUDONG GUO, ZHIXIONG YANG, GUANGHUA CHEN, WENMEI SU

**Affiliations:** 1 Department of Pulmonary Oncology, Affiliated Hospital of Guangdong Medical University, Zhanjiang, 524001, China; 2 Department of Thoracic Surgery, Affiliated Hospital of Guangdong Medical University, Zhanjiang, 524001, China; 3 Department of Orthopedics, Affiliated Hospital of Guangdong Medical University, Zhanjiang, 524001, China; 4 Guangdong Provincial Key Laboratory of Autophagy and Major Chronic Non-Communicable Diseases, Affiliated Hospital of Guangdong Medical University, Zhanjiang, 524001, China

In the article “Silencing of the long non-coding RNA LINC00265 triggers autophagy and apoptosis in lung cancer by reducing protein stability of SIN3A oncogene” (*Oncology Research*. 2024, Vol. 32, No. 7, pp. 1185–1195. doi: 10.32604/or.2023.030771, https://www.techscience.com/or/v32n7/57163), an inadvertent error occurred during the compilation of Fig. 3H. This needed corrections to ensure the accuracy and integrity of the data presented.

Issue with Fig. 3H:

- Original Issue: The original Fig. 3H contained an incorrect image. Due to confusion with the folder of another si*LINC00265*-2 image (Fig. 3H) during the image selection process, which led to the image for si*LINC00265*-1 was incorrectly placed. This resulted in a mismatch between the image and the actual experimental conditions it was intended to depict.
- Reason for Change: To accurately represent the apoptosis results under the specified experimental conditions (si*LINC00265*-1 group), we have revised Fig. 3H. The new image correctly labels the si*LINC00265*-1 and accurately corresponds to the intended experimental setup.
- Impact on Results: The revision of Fig. 3H does not involve any alteration of the legends or text associated with the figure. It replaces the erroneous image with the correct one, ensuring that the visual data accurately corresponds to the reported experimental findings. This correction does not impact the scientific conclusions drawn in the study.

The corrected versions of Fig. 3 is provided. The changes were necessary to maintain the integrity of the published work and to provide accurate visual data to support the study’s findings. The authors confirm that these corrections do not alter any of the study’s results or conclusions. This correction has been approved by the *Oncology Research* Editorial Office, and the original publication has been updated accordingly.

**Table 1:** The authors would like to correct the figure as follows

Page No.	Exact figure to be corrected	Correction
1190	Fig. 3	Replace with new Fig. 3

**Figure 3**

**Figure 3:**
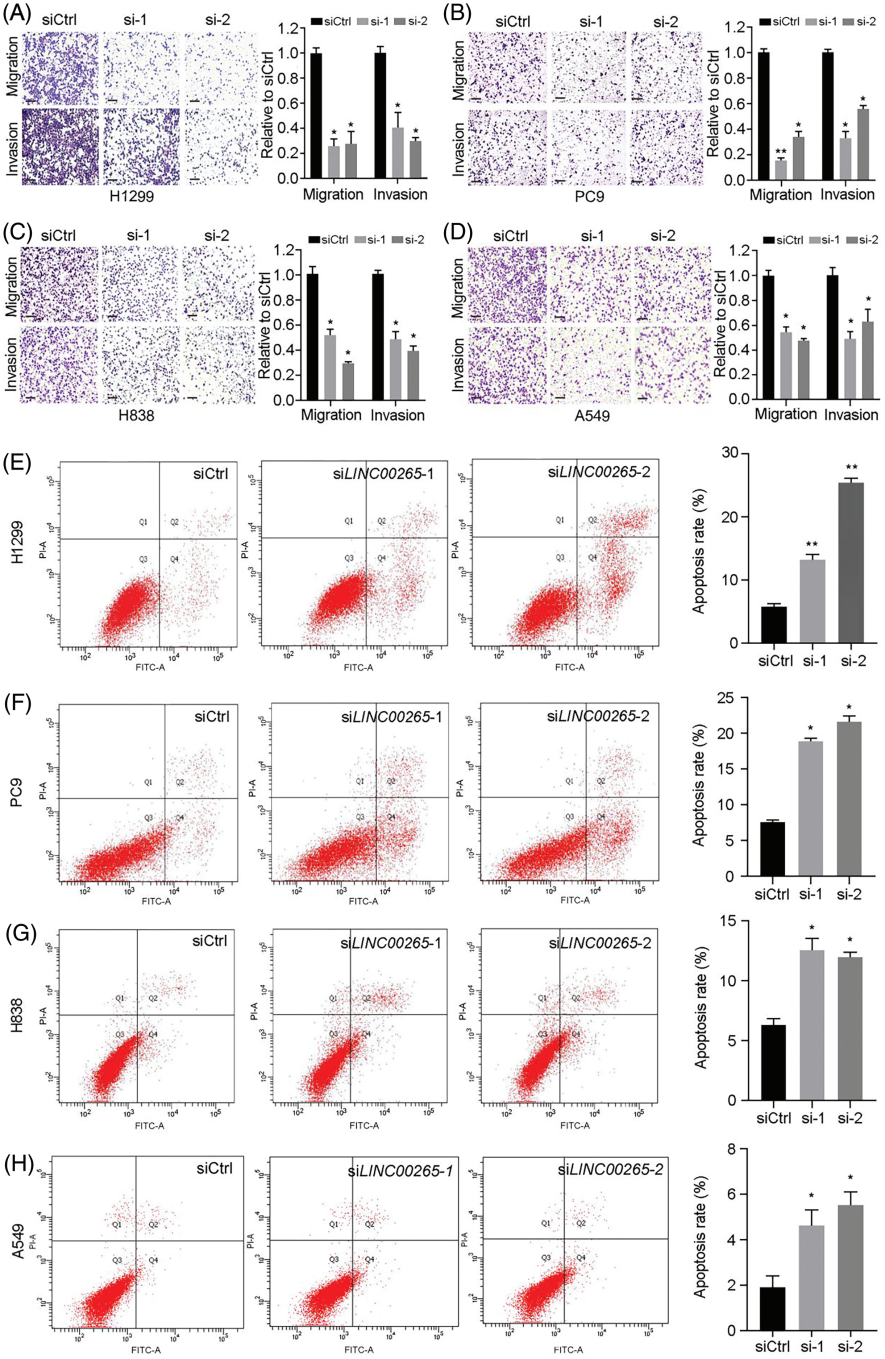
Silencing of *LINC00265* inhibits migration and invasion of NSCLC cells and induces apoptosis. (A–D) Transwell assays show the effects of *LINC00265* knockdown on NSCLC migration and invasion. The value in siCtrl-treated conditions is set to 1 as a reference. Data are the mean ± s.e.m. from three independent experiments (*p < 0.05; **p < 0.01). Scale bar, 100 μm. (E–H) Flow cytometry analyses show that knockdown of LINC00265 increases apoptosis rate in NSCLC cells (*p < 0.05; **p < 0.01).

