# Supplementary figures and images for: Silencing of the long non-coding RNA LINC00265 triggers autophagy and apoptosis in lung cancer by reducing protein stability of SIN3A oncogene
Source: Oncol Res. 2024 Jun 20;32(7):1185–95. doi: 10.32604/or.2023.030771 (PMC11211643; doi:10.32604/or.2023.030771)

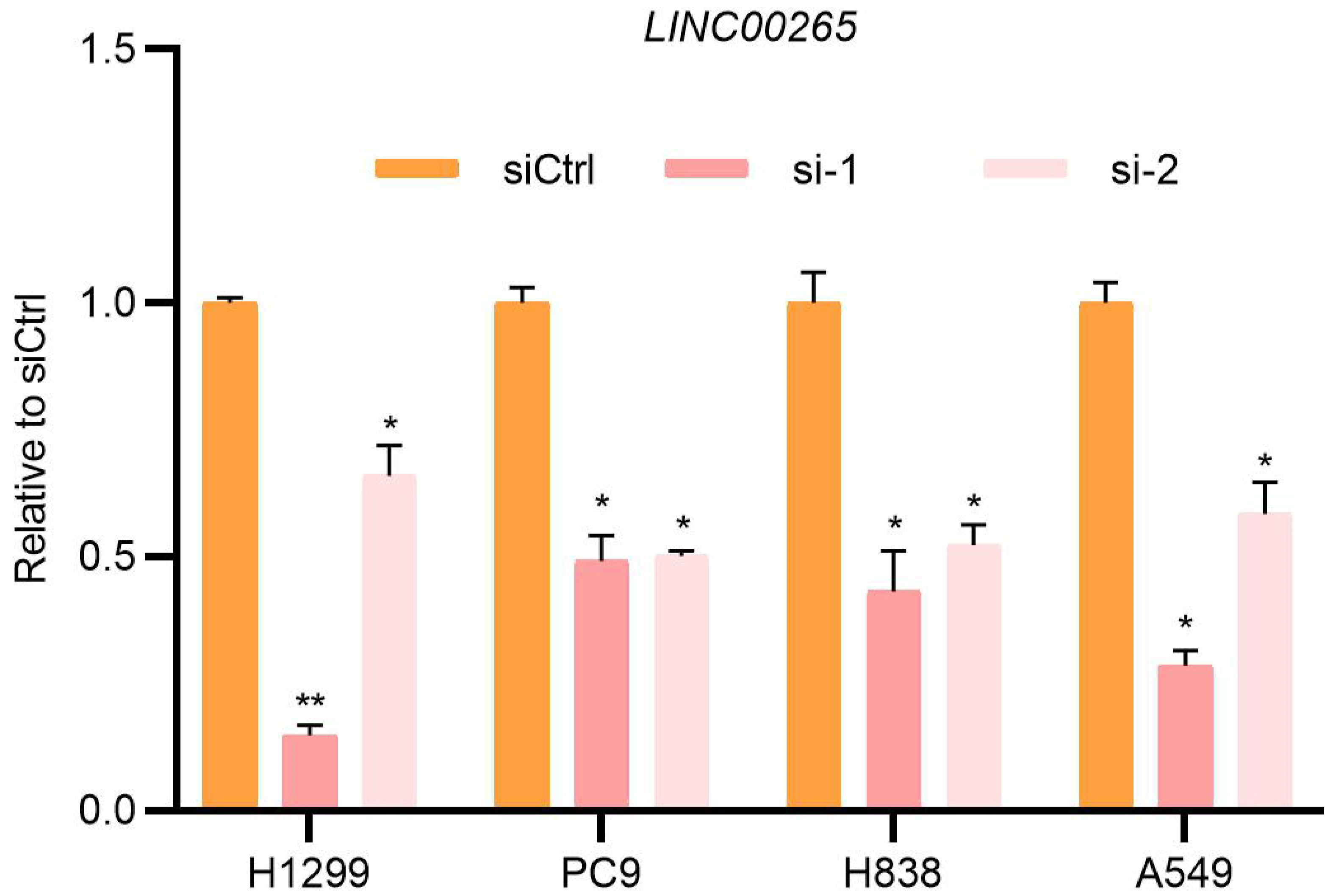

Supplement: Figure S1. [file OncolRes-32-30771-s001.tif]

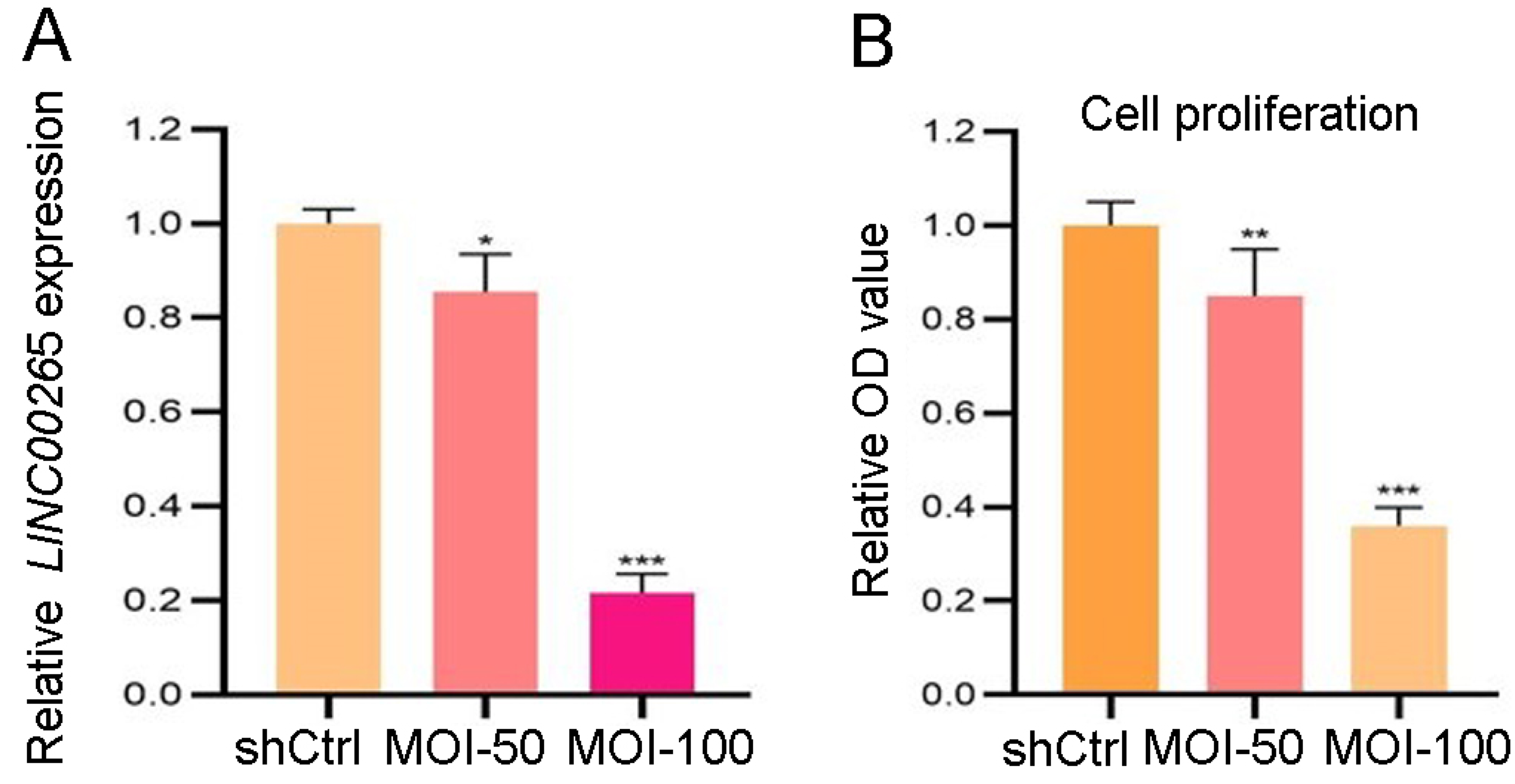

Supplement: Figure S2. [file OncolRes-32-30771-s002.tif]

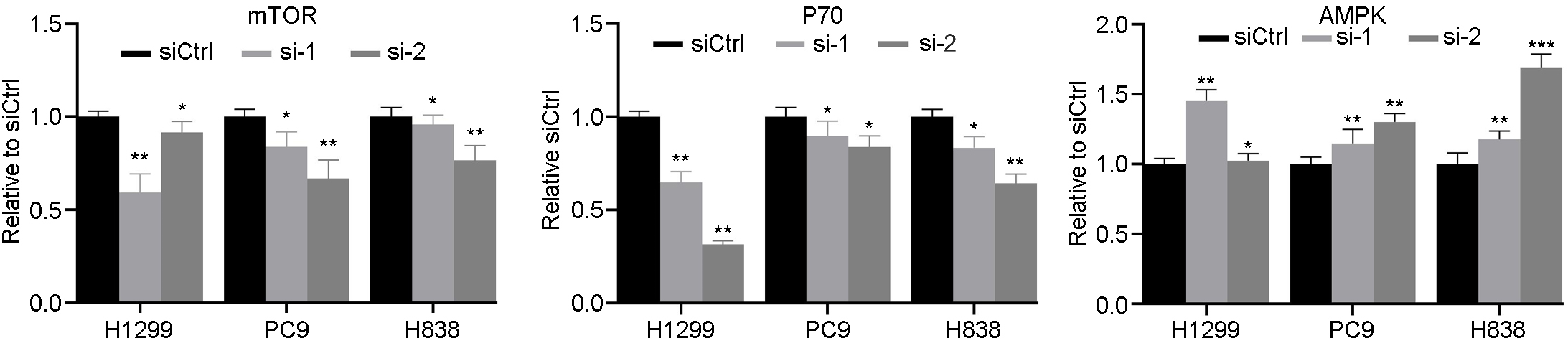

Supplement: Figure S4. [file OncolRes-32-30771-s004.tif]

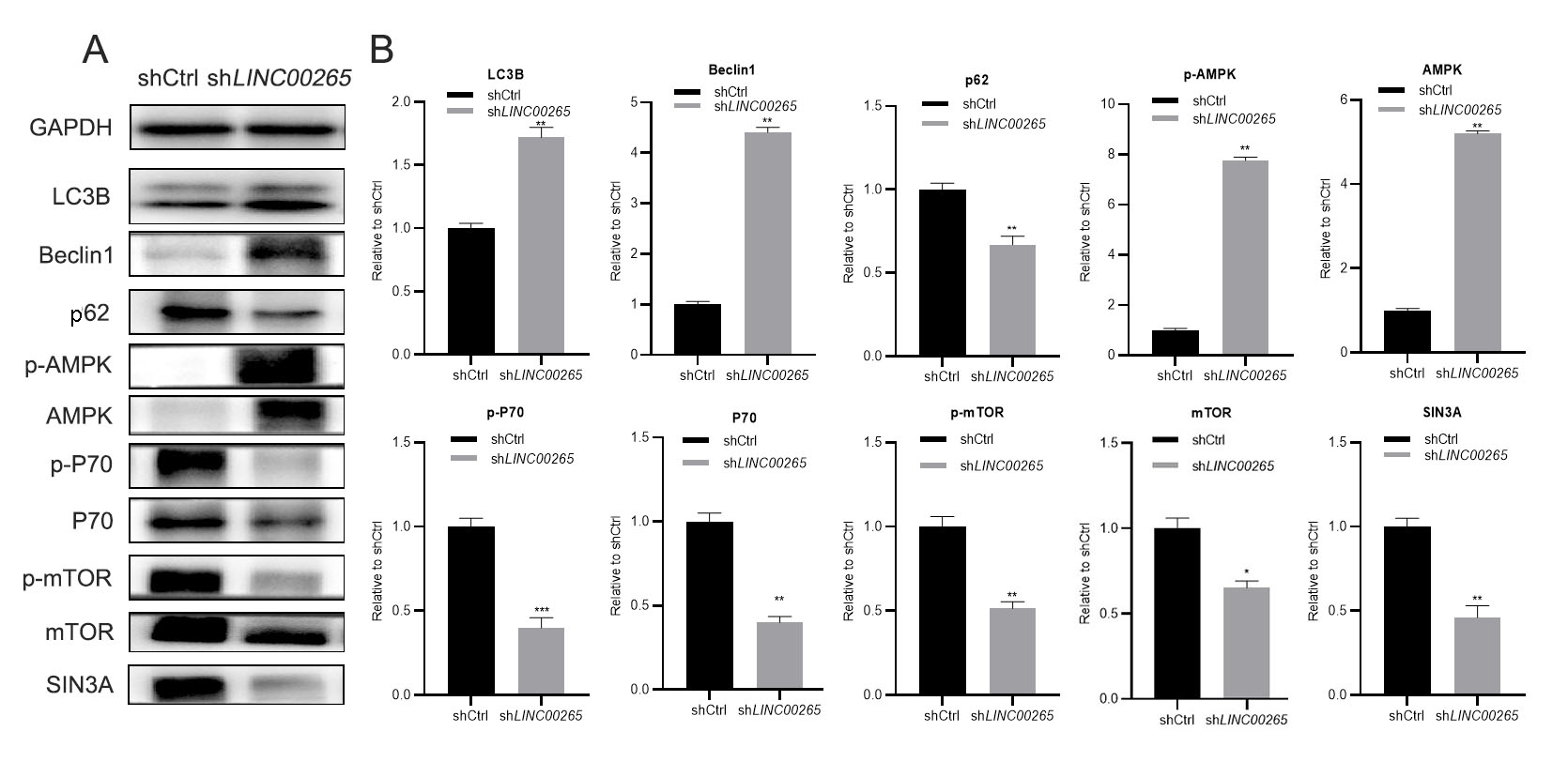

Supplement: Figure S5. [file OncolRes-32-30771-s005.tif]

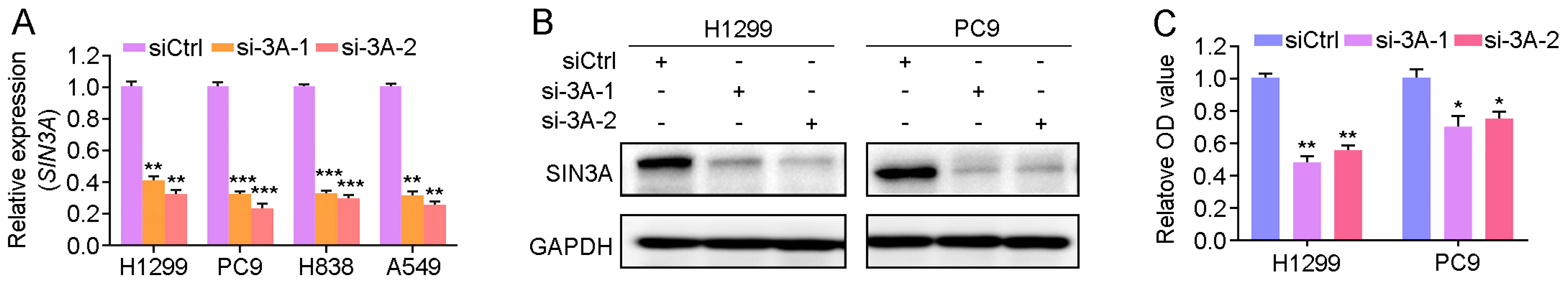

Supplement: Figure S6. [file OncolRes-32-30771-s006.tif]
