# Supplementary material for: Silencing of the long non-coding RNA LINC00265 triggers autophagy and apoptosis in lung cancer by reducing protein stability of SIN3A oncogene
Source: Oncol Res. 2024 Jun 20;32(7):1185–95. doi: 10.32604/or.2023.030771 (PMC11211643; doi:10.32604/or.2023.030771)
Supplement: Supplementary file 7 [file OncolRes-32-30771-s001.docx]

**Table S1. siRNA sequences used in this study.**

| siRNAs | Sequences |
| --- | --- |
| *LINC00265*#1 | 5’-CCUAGUCUCCUUCCCGCUUTT-3’  5’-AAGCGGGAAGGAGACUAGGTT-3’ |
| *LINC00265*#2 | 5’-GGGACCUCAACACAUUUGATT-3’  5’-UCAAAUGUGUUGAGGUCCCTT-3’ |
| SIN3A#1 | 5’-GCAGUCAGCUACGGGAAUUTT -3’  5’-AAUUCCCGUAGCUGACUGCTT -3’ |
| SIN3A#2 | 5’-CCUCAGGUCUACAAUGAUUTT -3’  5’-AAUCAUUGUAGACCUGAGGTT -3’ |
| siRNA negative control | 5’-UUCUCCGAACGUGUCACGUTT-3’  5’-ACGUGACACGUUCGGAGAATT-3’ |

**Table S2. PCR primer sequences used in this study.**

| Primer names | Primer Sequences (5’-3’) |
| --- | --- |
| *LINC00265*-F | ACAGGTCTCATGCTCTCACTCAGG |
| *LINC00265*-R | GCTACTCGGGAGGCTAAGTCAGG |
| SIN3A-F | CAGCTACGTCTCAAAGAACCTA |
| SIN3A-R | GCATGAATGGTGAACATCTCTC |
| GAPDH-F | GTCAAGGCTGAGAACGGGAA |
| GAPDH-R | AAATGAGCCCCAGCCTTCTC |
| U1-F | ACCTGGCAGGGGAGATACCA |
| U1-R | GAAAGCGCGAACGCAGTCC |

**Table S3. Antibodies used in this study.**

| Antibodies | Providers | Cat # |
| --- | --- | --- |
| GAPDH | Sigma | AB2302 |
| LC3B-II | abcam | ab192890 |
| p62 | Cell Signaling Technology | 5114 |
| Beclin1 | Cell Signaling Technology | 3495 |
| p-AMPK | Cell Signaling Technology | 2535 |
| AMPK | Cell Signaling Technology | 5832 |
| p-p70 | Cell Signaling Technology | 9234 |
| p70 | Cell Signaling Technology | 2708 |
| p-mTOR | Cell Signaling Technology | 5536 |
| mTOR | Cell Signaling Technology | 2983 |
| SIN3A | Cell Signaling Technology | 8056 |
| SNRNP70 | Millipore | CS203206 |
